# Enzymatic Phosphorylation of Oxidized Tyrosine Residues

**DOI:** 10.1021/acs.jproteome.3c00061

**Published:** 2023-05-05

**Authors:** Juho Heininen, Catharina Erbacher, Tapio Kotiaho, Risto Kostiainen, Jaakko Teppo

**Affiliations:** † Drug Research Program and Division of Pharmaceutical Chemistry and Technology, Faculty of Pharmacy, University of Helsinki, P.O. Box 56, FI-00014 Helsinki, Finland; ‡ Institute of Inorganic and Analytical Chemistry, University of Münster, Corrensstraße 48, 48149 Münster, Germany; § Department of Chemistry, Faculty of Science, University of Helsinki, P.O. Box 55, FI-00014 Helsinki, Finland

**Keywords:** phosphorylation, insulin receptor, oxidation−reduction (redox), post-translational modification (PTM), ultra-high-performance liquid chromatography (UHPLC), mass spectrometry (MS), liquid chromatography-tandem mass spectrometry (LC-MS/MS)

## Abstract

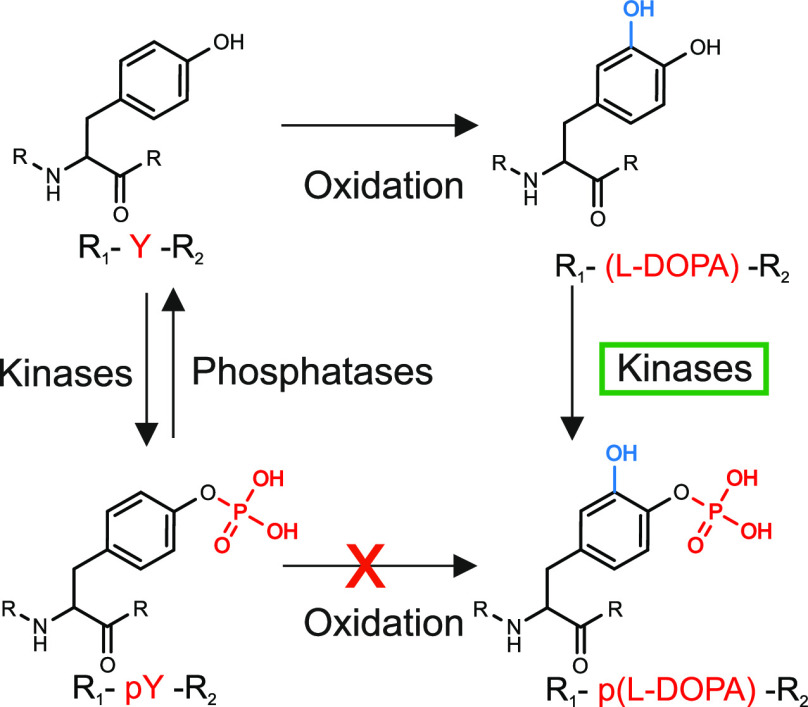

Post-translational modifications (PTMs) alter the function and fate of proteins and cells in almost every conceivable way. Protein modifications can occur as a result of specific regulating actions of enzymes, such as tyrosine kinases phosphorylating tyrosine residues or by nonenzymatic reactions, such as oxidation related to oxidative stress and diseases. While many studies have addressed the multisite, dynamic, and network-like properties of PTMs, only little is known of the interplay of the same site modifications. In this work, we studied the enzymatic phosphorylation of oxidized tyrosine (l-DOPA) residues using synthetic insulin receptor peptides, in which tyrosine residues were replaced with l-DOPA. The phosphorylated peptides were identified by liquid chromatography-high-resolution mass spectrometry and the site of phosphorylation by tandem mass spectrometry. The results clearly show that the oxidized tyrosine residues are phosphorylated, displaying a specific immonium ion peak in the MS^2^ spectra. Furthermore, we detected this modification in our reanalysis (MassIVE ID: MSV000090106) of published bottom-up phosphoproteomics data. The modification, where both oxidation and phosphorylation take place at the same amino acid, has not yet been published in PTM databases. Our data indicate that there can be multiple PTMs that do not exclude each other at the same modification site.

## Introduction

Protein phosphorylation catalyzed by kinases is a reversible post-translational modification (PTM) that has a central role in the regulation of cell functions. It is estimated that up to 75% of proteins encoded by the human genome can be phosphorylated.^1−3^ Most commonly, protein phosphorylation takes place at serine, threonine, and tyrosine residues, although noncanonical phosphorylation of other amino acid residues is possible.^4^ Multiple amino acid residues in a protein can be phosphorylated by two or more kinases, and multisite phosphorylation is now known to be typical, rather than an exception.^2^ Protein phosphorylation results in a structural conformational change in the protein that affects its function, acting as a switch, deactivating and activating enzymes and receptors, and thus providing flexible mechanisms for cells to respond to external stimuli. Abnormalities in phosphorylation can have pathogenic effects, as they disrupt the regulatory mechanisms and signaling pathways of cells.

Proteins in the human proteome are phosphorylated by 518 protein kinases and dephosphorylated by ∼200 protein phosphatases.^5,6^ Tyrosine phosphorylation is especially peculiar, as 90 of the 518 protein kinases are tyrosine kinases, but phosphorylated tyrosine (pTyr) residues constitute below 1% of protein phosphorylation in total, suggesting heavy and wide regulatory activity.^1,6−8^ Disruptions in signaling pathways occur in the pathophysiological mechanisms of many diseases, among them several types of cancer.^9^ Indeed, many tyrosine kinases show oncogenic activity, which can be caused either by mutation or by overexpression.^10^ Chronic myelogenous leukemia (CML) is a well-known example of a disease in which tyrosine kinases play a role.^11^ A remarkable step in treating this form of cancer was the development of different tyrosine kinase inhibitors to suppress the activity of the overactive enzymes, making CML, previously a lethal disease, treatable.^12^

Besides phosphorylation, protein oxidation can have a huge impact on cellular activity. Oxidative stress from endogenous (e.g., mitochondrial respiratory chain and aerobic exercise, hyperoxia, enzymatic reactions, immune response) and exogenous (e.g., pollutants, exposure to radiation, certain drugs, xenobiotics) sources forms reactive oxygen and nitrogen species (ROS and RNS, respectively).^13^ ROS and RNS can react with a large number of biomolecules such as lipids, carbohydrates, nucleic acids, and amino acid residues of proteins.^13^ Oxidative modifications of proteins can result in changes in their physical and chemical properties, including conformation, structure, solubility, susceptibility to proteolysis, and enzyme activity.^14^ Protein oxidation has been associated with various biological consequences, including aging and disorders such as Alzheimer’s and Parkinson’s diseases and amyotrophic lateral sclerosis (ALS).^15,16^ Several amino acids can be directly modified via side-chain reactions with ROS, but the most reactive amino acid residues are sulfur-containing amino acids (cysteine and methionine) and those with aromatic structures (e.g., phenylalanine, histidine, and tyrosine).

The oxidation of phosphorylated amino acid residues or phosphorylation of oxidized amino acid residues in proteins and peptides has rarely been investigated. The residues that are prone to both oxidation and phosphorylation are tyrosine and histidine. In proteins and peptides, tyrosine residues, which take part in signaling processes by phosphorylation,^17^ are especially prone to oxidation, resulting in protein-bound l-3,4-dihydroxyphenylalanine (l-DOPA) as well as other products.^18^ Ruokolainen et al. have demonstrated that tyrosine oxidation is inhibited after the initial phosphorylation of the tyrosine residues.^19^ Gow et al. showed that peroxynitrite-mediated nitration of tyrosine residues significantly inhibits the phosphorylation of a synthetic peptide by a tyrosine kinase.^20^ It has been suggested that besides direct oxidative damage to biomolecules, cell toxicity by oxidation could be partially induced by the inhibition of tyrosine phosphorylation and the consequent interference with cellular signaling.^17^ Similarly, Zhang et al. reported the first proteome-wide survey of endogenous site-specific tyrosine oxidation to DOPA modifications in mouse heart and brain tissues. Many of the oxidation sites were also possible phosphorylation sites, and it was hypothesized that this oxidation would disrupt tyrosine phosphorylation signaling pathways.^21^

**Figure 1:**
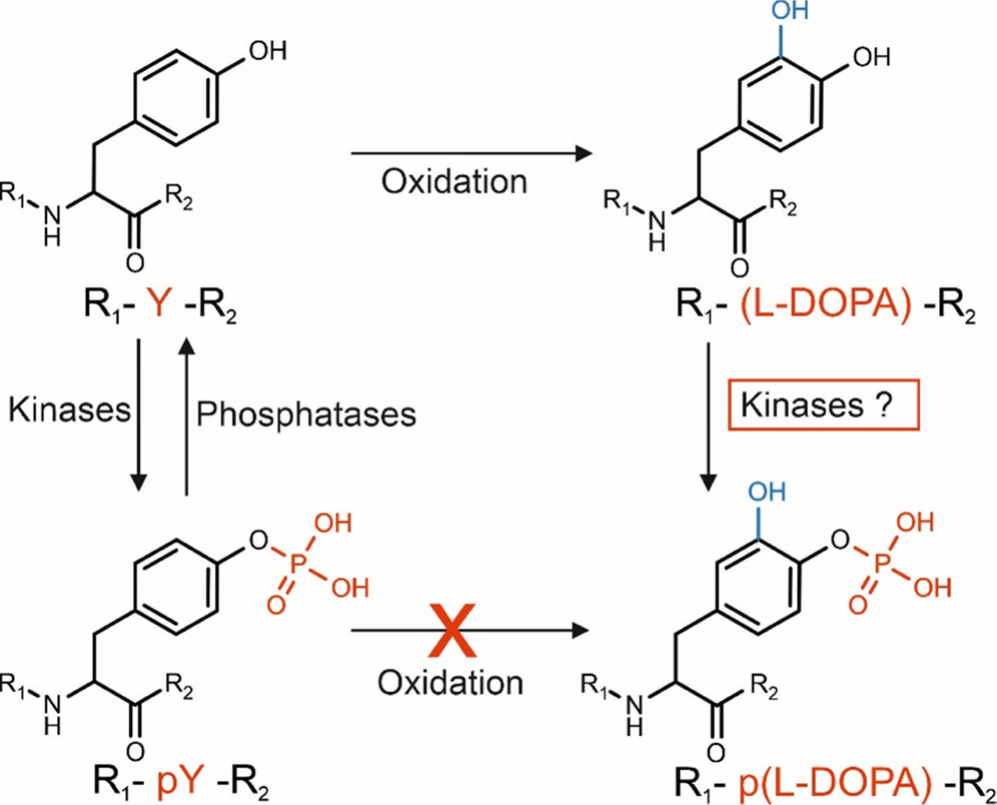
Structures and formation relationships of tyrosine, l-DOPA, phosphotyrosine, and phospho-l-DOPA. Tyrosine residues are phosphorylated by kinases and dephosphorylated by phosphatases. Phosphorylated tyrosine residues have been shown to be unsusceptible to oxidation.^19^ Tyrosine residues can also be oxidized to, e.g., l-DOPA. Kinase-catalyzed phosphorylation of l-DOPA residues has not been reported previously.

Recently, in their research on proteome-wide PTM mapping in human hearts, Bagwan et al. reported peptides with a mass change of 95.961 Da, which they attributed to a combination of phosphorylation and oxidation in the same peptide, but modification on the same amino acid residue was not considered.^22^ So far, the phosphorylation of l-DOPA residues formed by oxidation of tyrosine has not been studied (Figure 1).

The aim of this work was to evaluate the phosphorylation of l-DOPA residues of synthetic insulin receptor peptides IR0, IR1, IR2, and IR3 (Table 1) by a tyrosine kinase. The peptide sequence corresponds to amino acid residues 1142–1153 of the insulin receptor β-subunit cytoplasmic domain, which includes the regulatory autophosphorylation sites Tyr1146, Tyr1150, and Tyr1151. IR0 included three tyrosine residues, IR1 one l-DOPA and two tyrosine residues, IR2 two l-DOPA and one tyrosine, and IR3 three l-DOPA residues. The phosphorylation of the tyrosine and l-DOPA residues was examined by liquid chromatography-mass spectrometry (LC-MS) using an Orbitrap mass spectrometer. Because the phosphorylated l-DOPA is not published in the UniMod database,^23^ the presence of phosphorylated and unphosphorylated l-DOPA in published raw phosphoproteomics data was also searched using variable modification search and unrestricted modification search.

**Table 1:** Amino Acid Sequences of the IR Peptides

peptide	sequence
IR0	TRDI**Y**ETD**YY**RK
IR1	TRDI(**l****-DOPA**)ETD**YY**RK
IR2	TRDI(**l****-DOPA**)ETDY(**l****-DOPA**)RK
IR3	TRDI(**l****-DOPA**)ETD(**l****-DOPA**)(**l****-DOPA**)RK

## Experimental Procedures

### Peptides and Reagents

Peptide IR0 was from Designer BioScience Ltd. (Cambridge, U.K.), and synthetic peptides IR1, IR2, and IR3 were from CASLO ApS (Kongens Lyngby, Denmark). All peptides have the same sequence TRDIYETDYYRK with a varying number of oxidized tyrosine residues (i.e., l-DOPA). In IR0, all tyrosine residues are nonoxidized. In IR1 Tyr5 residue, in IR2 Tyr5 and Tyr10 residues, and in IR3, all of the tyrosine residues (Tyr5, Tyr9, and Tyr10) are oxidized (Table 1). The producer-reported purities of the peptides were over 96% as determined by LC-MS.

Formic acid was purchased from Merck (Darmstadt, Germany), 5× kinase buffer A from Thermo Fisher (Bremen, Germany), adenosine triphosphate (ATP) from Sigma-Aldrich (Steinheim, Germany), and IR kinase (INSR kinase, GST-tagged human recombinant protein PR7080A) from Life Technologies (Carlsbad, CA). LC-MS grade methanol and acetonitrile (Honeywell Riedel-de Haën, Bucharest, Romania) were used as solvents. Deionized water was prepared with a Milli-Q water purification system (Milli-Q Integral 15 Water Purification System with Quantum TEX cartridge) on site.

### Samples and Phosphorylation Reactions

1× kinase buffer A was prepared by dilution with Milli-Q water from the 5× commercial stock solution. 1 mg mL^–1^ (0.6 mM) of individual peptide solutions of IR0, IR1, IR2, and IR3 and 5 mg mL^–1^ (9.1 mM) of ATP solution were separately prepared in 1× kinase buffer A. 20 μL of IR kinase (0.39 mg mL^–1^, 5.45 nM) was diluted to 0.1 mg mL^–1^ (1.4 nM) by adding 58 μL of 1× kinase buffer A. Samples for kinase incubation were made according to Table S1. After mixing, incubation was carried out at 37 °C in a warm-air oven with shaking for 3 h, excluding the samples for the investigation of the reaction rate (1, 5, 30, and 60 min incubation). After incubation, the reaction was quenched using 3 μL of 100% formic acid, followed by evaporation to dryness with a vacuum centrifuge (SpeedVac). Dry samples were reconstituted for LC-MS analysis by adding 40 μL of 1% methanol and 0.1% formic acid in water (LC-eluent A). The samples were sonicated in an ultrasonic water bath at room temperature for 15 min and transferred to autosampler vials.

### LC-MS and LC-MS/MS Acquisition

The LC-MS measurements were carried out using an Orbitrap Fusion tribrid mass spectrometer coupled to an UltiMate 3000 liquid chromatography setup (both from Thermo Fisher Scientific). Chromatography was performed using a Waters Acquity UPLC C-18 column (HSS T3, 2.1 mm × 100 mm, 1.7 μm with an inline filter) at 30 °C. The autosampler temperature was 15 °C, and the injection volume was 3 μL. Eluent A consisted of 1% methanol and 0.1% formic acid in water in positive ion mode and 3% methanol and 0.1% formic acid in negative ion mode, while eluent B consisted of 0.1% formic acid in methanol in both measurements. The flow rate was 0.29 mL min^–1^. The gradient started from 0% B and was then increased to 50% B in 8 min, to 95% B within the next 2 min, and held at 95% B for 3.5 min. After that, the gradient was decreased to 0% B within 0.5 min. Subsequently, a cleaning step followed, where the gradient was increased to 95% B in 1 min and after that decreased back to 0% in 1 min. The gradient was held at 0% B for 4 min, resulting in a total gradient time of 20 min.

Mass spectra were measured using electrospray ionization with a 3.5 kV spray voltage in positive ion mode and a −2.5 kV spray voltage in negative ion mode. The orbitrap resolution was 500 000 (at *m/z* 200), and the scan range was *m/z* 150–1500. Automated gain control (AGC) was set to accumulate 1 × 10^5^ ions with a maximum injection time of 100 ms, and the RF lens was set to 60%. The ion transfer tube temperature was 325 °C, and the vaporizer temperature was set to 350 °C. Internal mass calibration with Easy-IC (fluoranthene) was used.

MS/MS measurements were performed using a data-dependent acquisition method (DDA) with an inclusion list of possible precursor ions from [M + 3H]^3+^ peptides (exact masses in Table S2). DDA consisted of an MS^1^ scan with a mass range of *m/z* 50–1000 and a resolution of 120 000, followed by MS/MS scans for the cycle time of 2 s with a quadrupole isolation window of 1.1 Da, collision-induced dissociation (CID) fragmentation with a fixed collision energy of 30%, an orbitrap resolution of 60 000, and a scan range of *m/z* 50–1000. For MS/MS, AGC was set to accumulate 1 × 10^5^ ions with a maximum injection time of 118 ms.

Average MS/MS spectra of the main phosphorylation products were annotated individually for peptide-specific a, b, y, and x fragments and neutral losses of NH_3_ or H_2_O and verified using interactive peptide spectral annotator (IPSA)^24^ for fragments with relative abundance above 1%. To distinguish the possible loss of phosphorylation during the MS/MS scan, all mass spectra were reviewed against nonphosphorylated controls using IPSA. Modifications added to the unmodified peptide sequence in IPSA were +15.9949 Da for tyrosine oxidation to l-DOPA, +79.9663 Da for phosphorylation, and +95.9612 Da for both phosphorylation and oxidation in the same amino acid.

### Analysis of Phosphoproteomics Data

Raw phosphoproteomics data^1,25−29^ were downloaded from the MassIVE repository and obtained from collaborators. Protein identification was done with MaxQuant/Andromeda (MaxQuant 2.1.0.0 and 2.1.4.0).^30−32^ The search database used was reviewed human or mouse UniProt/Swiss-Prot^33^ proteome (20 385 or 17 053 entries, respectively; FASTA files downloaded on 17 November 2020). A combination of phosphorylation and oxidation (phospho-oxidation) in the same tyrosine residue was searched using both a dependent peptide (DP) search (that does not require *a priori* assumptions of the PTMs) and a standard variable modification search method. Methionine oxidation, serine/threonine/tyrosine phosphorylation, tyrosine oxidation, and tyrosine phospho-oxidation (added to MaxQuant modification table with P(1) O(4) H(1) composition resulting in a mass change of +95.9612 Da) were set as variable modifications. Cysteine carbamidomethylation was set as a static modification. DP search was performed alongside the variable modification search using a DP-specific FDR of 0.01. The largest data set^1^ was rerun for DP without the same site tyrosine phospho-oxidation as a variable modification to remove these peptide spectral matches from the main or second peptide search and thus permitting unassigned MS/MS spectra to be matched in dependent peptide search. This DP reanalysis was done using cysteine carbamidomethylation as a static modification and methionine oxidation and serine/threonine/tyrosine phosphorylations as variable modifications.

Modification site and dependent peptide tables were used for data analysis. Decoy database matches and potential contaminants were removed. DP results were parsed to residue locations, location probability, and individual PSM. DP results containing “peptide:” prefix in annotated modification column were removed. Stringent identification and filtering criteria were applied: false discovery rate (FDR) <0.01 was applied for both peptide and protein identification, and only modification sites with a localization probability of above 0.999 and posterior error probability (PEP) <0.01 were considered reliable identifications in the variable modification search. DP result bins with the highest localization on tyrosine were parsed to their individual peptide spectrum matches. The known diagnostic phosphorylated tyrosine immonium ion (C_8_H_11_NO_4_P^+^, *m/z* 216.04202) and the novel phosphorylated l-DOPA immonium ion (C_8_H_11_NO_5_P^+^, *m/z* 232.03694) reported in this work were searched. The quantification method (label-free/TMT/iTRAQ) was chosen according to the technique used in each study. Besides these, no changes were made to the recommended search settings.

### Experimental Design and Statistical Rationale

Each sample used for the verification of l-DOPA phosphorylation (tyrosine phospho-oxidation) is shown in Table S1, and samples in phosphoproteomics search results are in Table 3. The verification of l-DOPA phosphorylation was performed with IR peptides individually and as a mixture (Table S1, samples 7–15). Relative comparison of reaction rate was done using IR peptide mixture samples with 1, 5, 30, and 60 min incubation times (Table S1, samples 7–10). l-DOPA phosphorylation results were revised against matrix-matched control samples of eluent, blank with kinase, and peptides without kinase individually and as a mixture (Table S1, samples 1–6). Furthermore, the reagent ratios were optimized, and phosphorylation was replicated with additional standards and phosphorylated standard, individual, and mixture of IR peptides (similar to samples 1–15 in Table S1). These also serve as process replicates and complement LC-MS and LC-MS/MS results. Data analysis was performed as described above, and Microsoft Excel was used for statistical analysis.

Raw phosphoproteomics data sets were chosen to represent different sample types (human, *in vivo*, and *in vitro*), quantification methods (label-free and label-based), and research questions. In total, 223 raw files were searched. Experiment designs and the numbers of samples, replicates, and controls are described in the respective publications. No quantitative analysis was performed on the modified peptides that were identified.

### Data Availability

The original phosphorylated l-DOPA peptide mass spectra and the reanalyzed phosphoproteomics results are publicly available in the MassIVE repository (http://massive.ucsd.edu) with the identifier MSV000090106. Raw data for tyrosine oxidation and tyrosine phospho-oxidation in published phosphoproteomics results may be, according to Table 3, downloaded from ProteomeXchange^34^ or MassIVE, or inquired from original authors.^1,25−29^

## Results

### LC-MS

**Figure 2:**
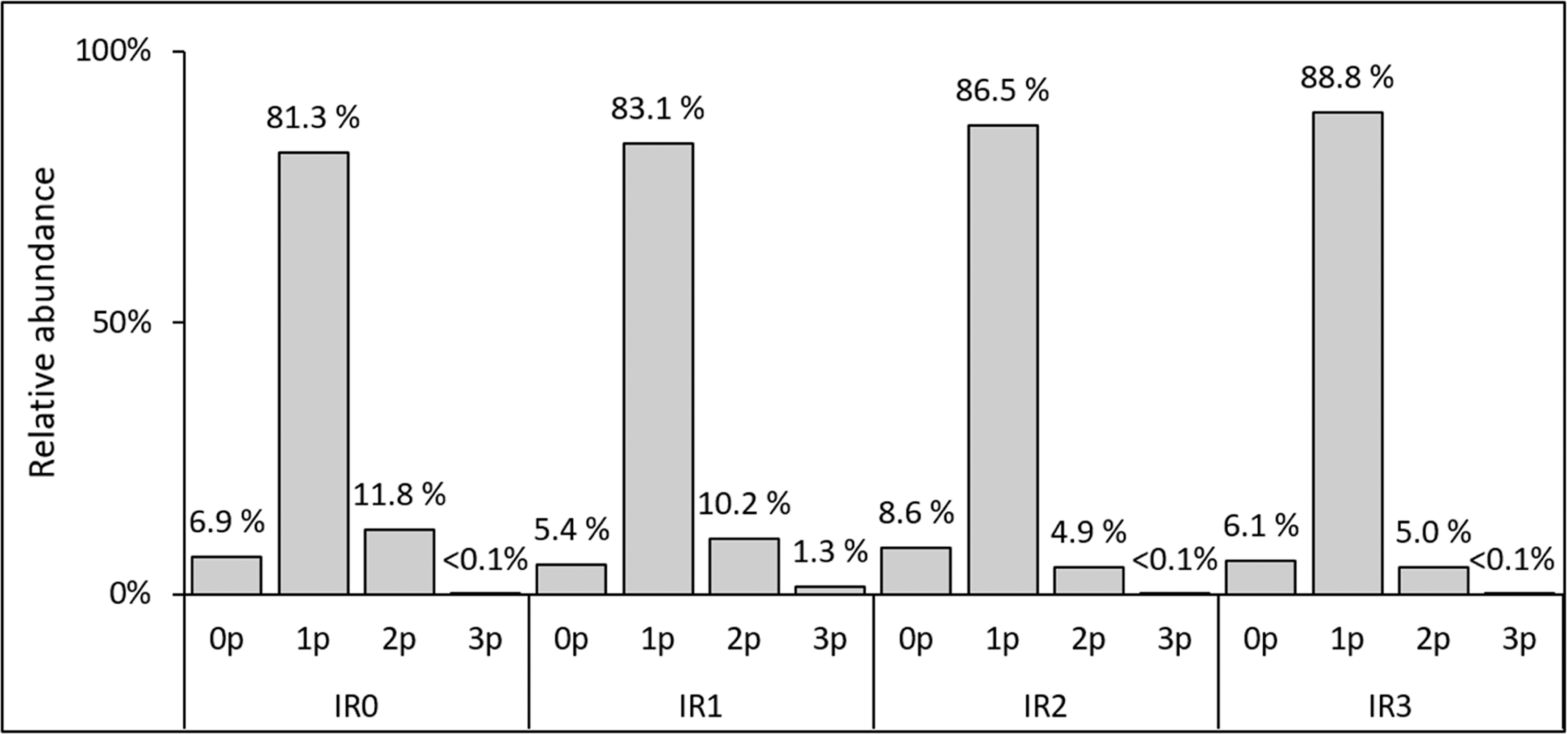
Relative abundances of phosphorylation products. Relative abundances of the nonphosphorylated and singly, doubly, and triply phosphorylated IR peptides formed in the enzymatic reactions shown with the most abundant analyte ion, [M + 3H]^3+^.

Phosphorylation of the differently oxidized tyrosine residues within the IR peptide analogs was studied using liquid chromatography-high-resolution mass spectrometry (LC-HRMS). IR0 includes three tyrosine residues, which in the other peptides were replaced with one (IR1), two (IR2), or three (IR3) l-DOPA residues (Table 1). The extracted ion chromatograms (EICs) of the cumulated multiply charged ions (+2 to +4 at positive ion mode, −2 only at negative ion mode) of the phosphorylation products of IR0, IR1, IR2, and IR3 are presented in Figures S1 and S2, respectively. The phosphorylation products were chromatographically separated with the elution order of decreasing polarity of the product, i.e., triply, doubly, and singly phosphorylated IR peptides. The nonphosphorylated IR peptide (starting material) eluted typically after the phosphorylated peptides. The EICs of the phosphorylation products (Figures S1 and S2) show that the main product was the singly phosphorylated product for each of the IR peptides (Figure 2). The relative abundances of doubly and triply phosphorylated products were below 12 and 2%, respectively, of all measured phosphorylation states (Figure 2). Only small amounts (<9% relative abundance) of nonphosphorylated IR peptides (starting material) were detected, indicating successful and efficient phosphorylation under the used reaction conditions.

Tables S2–S4 present the high-resolution positive and negative ion mass spectra of the phosphorylation reaction products with accurate masses, mass errors, and relative intensities. All mass spectra featured a charge state distribution from [M + 2H]^2+^ to [M + 4H]^4+^, the [M + 3H]^3+^ ion being the most intensive in positive ion mode (Figure S3) and [M – 2H]^2–^ the only ion in negative ion mode. The mass accuracies of the multiply charged ions were below 2.6 mmu, and those of the deconvoluted protonated and deprotonated molecules were below 5.2 mmu, with all phosphorylation products of IR0, IR1, IR2, and IR3, confirming the identification of the products.

**Figure 3:**
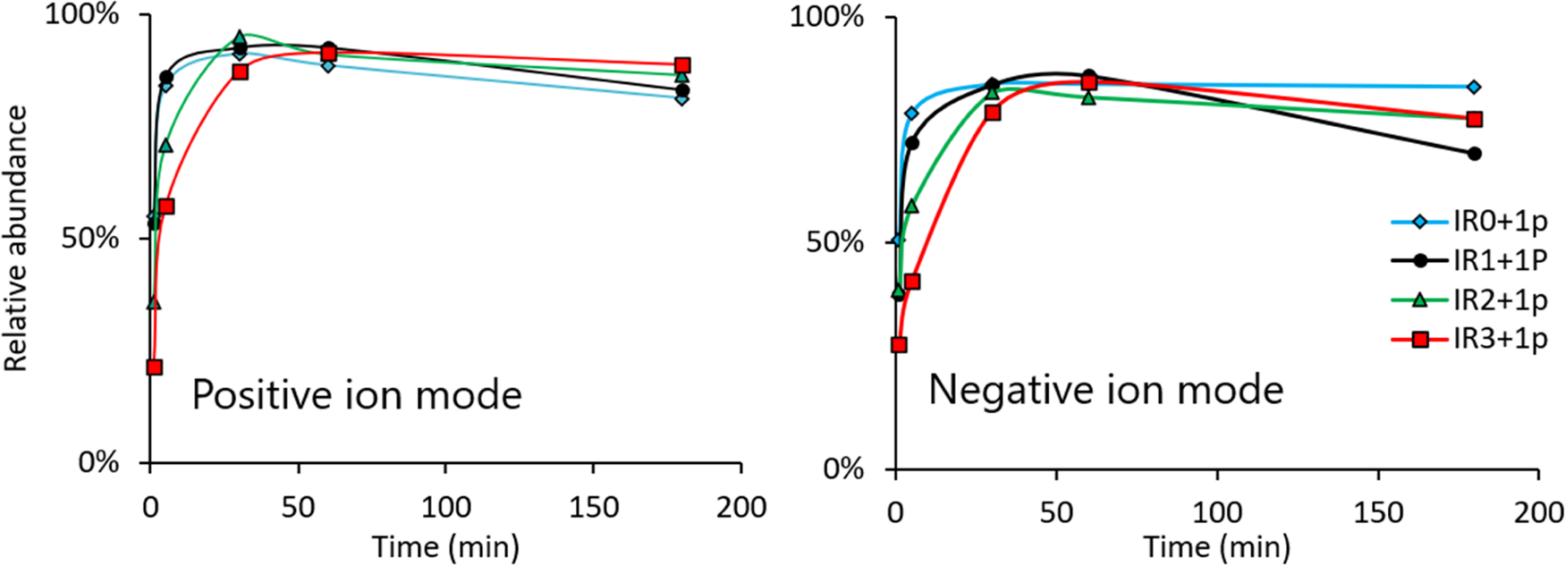
Formation of the singly phosphorylated IR peptides as a function of time. The relative abundance of singly phosphorylated peptides was measured using positive and negative ion modes. Relative abundance is the absolute intensity of IR + 1p divided by the absolute intensity of [(IR + 0p) + (IR + 1p) + (IR + 2p) + (IR + 3p)].

Because the singly phosphorylated product was the most abundant with all IR peptides, we studied the formation of singly phosphorylated IR peptides as a function of time using positive and negative ion modes (Figure 3). The results show that when all three tyrosines were oxidized to l-DOPA (i.e., IR3), the formation of singly phosphorylated IR3 (IR3 + 1p) was clearly slower than with IR0 (IR0 + 1p), in which none of the three tyrosines is oxidized. Also, the formation of IR2 + 1p was slower than the formation of IR0 + 1p. However, it is clear that oxidized tyrosine (l-DOPA) was phosphorylated but at a slightly lower rate than tyrosine.

### Determination of Phosphorylation Sites by LC-MS/MS

**Figure 4:**
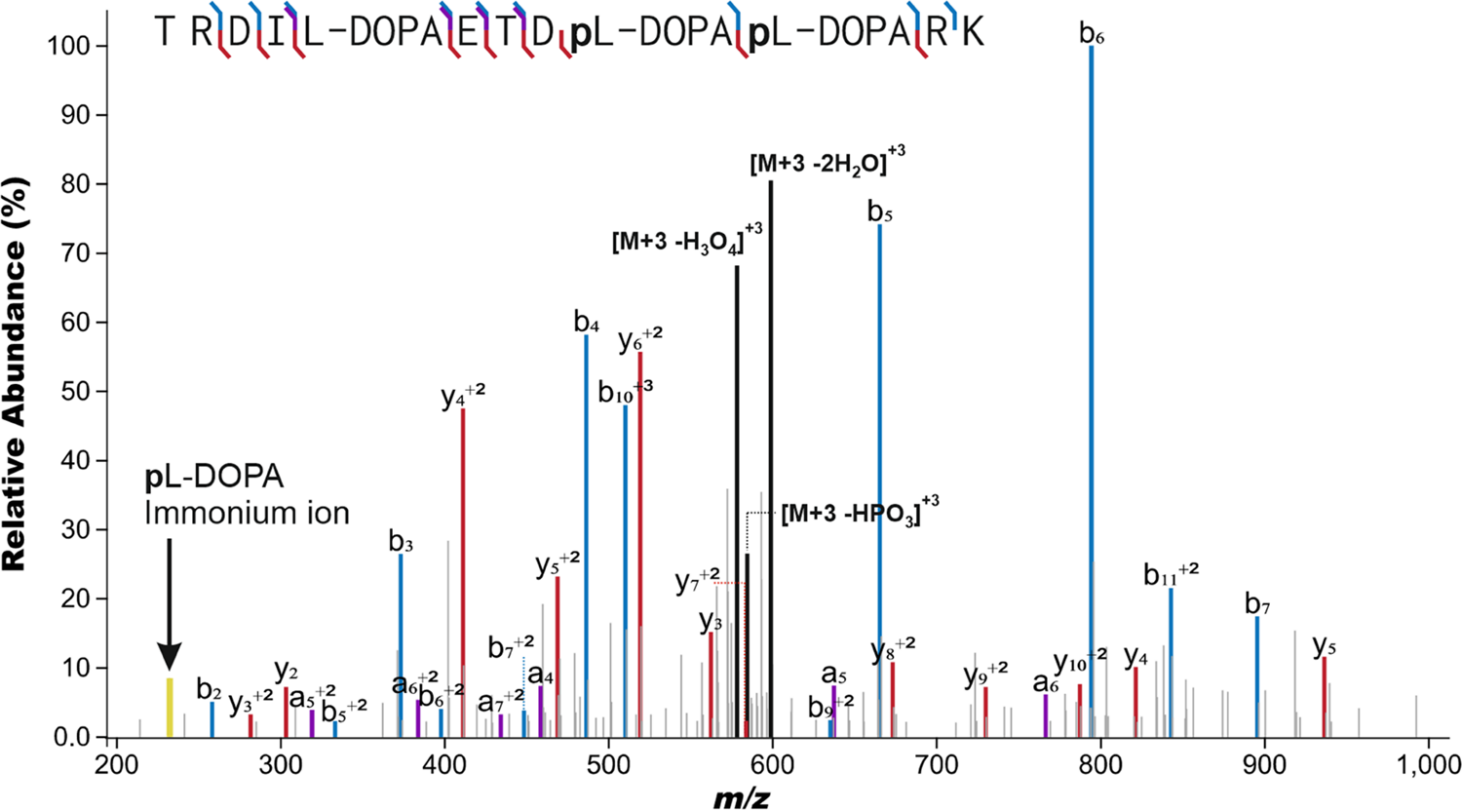
Example of an annotated MS/MS spectrum of doubly phosphorylated l-DOPA peptide IR3 + 2. IPSA^24^ was used to annotate the MS/MS spectrum of IR3 + 2p peptide, which has Tyr5 modification of +15.995 Da (oxidation) and both Tyr9 and Tyr10 with a modification of +95.961 Da (oxidation and phosphorylation). Only peaks above 2% relative abundance were annotated, and no neutral losses of, e.g., water, ammonia, or phosphate were annotated. Complete MS/MS spectrum annotations are in File S1.

LC-MS/MS was used to verify phosphorylation sites of the IR peptides. As the [M + 3H]^3+^ ions were the most abundant for all nonphosphorylated and phosphorylated IR peptides (Figure S3), they were selected as the precursor ions for the MS/MS analyses. No major coeluting and coisolating impurities were present in total ion chromatograms of LC-MS/MS (Figure S4) or in LC-HRMS data (Figures S1 and S2). The annotated MS/MS spectra of the main LC-MS/MS chromatographic peaks with accurate masses, mass errors, and relative abundances are presented fully in File S1 and as a representative exemplary spectrum in Figure 4.

The most common product ions were sequence diagnostic singly protonated N-terminal b ions and C-terminal y ions with charge states from +1 to +3 and those formed by the neutral loss of H_2_O and NH_3_. Also, the known phosphorylated tyrosine immonium ion (pY immonium ion, *m/z* 216.042) was observed with phosphorylated tyrosine (<0.5 ppm mass accuracy and 10.4% average relative abundance), and a novel phosphorylated l-DOPA immonium ion (pl-DOPA immonium ion, *m/z* 232.037) was observed specifically in the MS/MS spectra of peptides containing phosphorylated l-DOPA (<1.1 ppm mass accuracy and 4.6% average relative abundance, present in Figure 4). Tyrosine or l-DOPA phosphorylation site-specific b_4_ to b_5_ and y_3_ to y_5_ fragments are summarized in Table 2 and presented in detail in Table S5 with accurate masses, mass accuracies, and relative intensities.

**Table 2:** Diagnostic b and y Ions of Phosphorylated IR Peptides^b^

product	phosphorylation site	*t*_R_, min	precursor [M + 3H]^3+^	b_4_	b_5_	y_3_	y_4_	y_5_	immonium ion
IR0		5.99	541.6	X	X	X	X	X	
IR0 + p	Tyr9	5.89	568.3	X	X	X	X/p	X/p	pY
IR0 + 2p	mostly Tyr9 + Tyr10	5.65	594.9	X	X/p	X/p	p/2p	p/2p	pY
IR0 + 3p	Tyr5 + Tyr9 + Tyr10	5.19	621.6	X	X/p	p	p/2p	p/2p	pY
IR1		5.71	546.6	X	X	X	X	X	
IR1 + p	Tyr9	5.60	573.6	X	X	X	X/p	X/p	pY
IR1 + 2p	mostly Tyr9 + Tyr10	5.30	600.2	X	X	p	p/2p	p/2p	pY and pl-DOPA
IR1 + 3p	Tyr5 + Tyr9 + Tyr10	5.17	626.9	X	X/p	p	2p	2p	pY and pl-DOPA
IR2		5.50	552.3	X	X	X	X	X	
IR2 + p	Tyr9	5.37	578.9	X	X	X	X/p	X/p	pY
IR2 + 2p	mostly Tyr9 + Tyr10	5.30	605.6	X	X	X/p	p/2p	2p	pY and pl-DOPA
IR2 + 3p	Tyr5 + Tyr9 + Tyr10	5.15	632.2	X	p	a	2p	2p	
IR3		5.24	557.6	X	X	X	X	X	
IR3 + p	Tyr9	5.30	584.2	X	X	X	X/p	X/p	pl-DOPA
IR3 + 2p	mostly Tyr9 + Tyr10	5.27	610.9	X	X/p	X/p	p/2p	X/p/2p	pl-DOPA
IR3 + 3p	Tyr5 + Tyr9 + Tyr10	5.13	637.6	X	p	p	p/2p	p/2p	pl-DOPA

a Not observed.

b Nonphosphorylated fragments are marked with X, singly phosphorylated fragments with p, and doubly phosphorylated fragments with 2p. Present phosphotyrosine immonium ion is marked as pY and phospho-l-DOPA immonium ion as pl-DOPA. Accurate masses, relative abundances, and mass accuracies are provided in Table S5.

The MS/MS spectra of the main chromatographic peaks of singly, doubly, and triply phosphorylated IR peptides show very similar sites of phosphorylation (Table S5). The product ions y_4_ and y_5_ of singly phosphorylated peptides (IR0 + p, IR1 + p, IR2 + p, and IR3 + p) include a phosphate group, but y_3_ does not, indicating that the main site of phosphorylation is Tyr9 and l-DOPA9 in the case of IR3 + p. This is supported by the fact that neither tyrosine nor l-DOPA of the singly phosphorylated IR peptides at position 5 is phosphorylated, as phosphorylated b_5_ ions are not present. The MS/MS spectra of the doubly phosphorylated IR peptides (IR0 + 2p, IR1 + 2p, IR2 + 2p, and IR3 + 2p) show y_3_ with one phosphate group and both y_4_ and y_5_ ions with two phosphate groups, indicating that the residues Tyr9 and Tyr10 of IR0 and IR1, Tyr9, and l-DOPA10 of IR2, and l-DOPA9 and l-DOPA10 of IR3 are phosphorylated. The MS/MS spectra of all triply phosphorylated IR peptides (IR0 + 3p, IR1 + 3p, IR2 + 3p, and IR3 + 3p) show doubly phosphorylated y_4_ and y_5_ ions and singly phosphorylated b_5_ ions, indicating that all tyrosine or l-DOPA residues are phosphorylated. The IR peptides also show small abundances of different phosphorylation combinations (Tyr and l-DOPA), but these are not typically chromatographically separated or abundant enough for confident annotation. Furthermore, small abundances (below 5% relative abundance; Table S5) of corresponding nonphosphorylated sequence diagnostic b ions and y ions besides the phosphorylated diagnostic ions were observed. Examples of these are y_4_ and y_5_ ions in Table S5: singly phosphorylated peptides show singly phosphorylated fragments with a high relative abundance and unphosphorylated fragments with low relative abundance. Likewise, doubly phosphorylated peptides show doubly phosphorylated fragments with a high relative abundance and singly phosphorylated fragments with low relative abundance. These are likely caused by typical phosphate neutral loss, as peptide phosphorylation is a labile modification.^35^

LC-MS/MS results confirm that up to three phosphorylations take place on all three tyrosine or l-DOPA residues. No other amino acid of the studied IR peptides was phosphorylated, and no l-DOPA was phosphorylated twice. The earlier results^19^ show that tyrosine residues in peptides and proteins can be oxidized to l-DOPA, and the results in this work show that l-DOPA residues in peptides can be phosphorylated by a tyrosine kinase.

### Phosphorylation of Oxidized Tyrosine Residues in Phosphoproteomics Data

Subsequently, published phosphoproteomics data^1,25−29^ was used to investigate whether tyrosine oxidation, alone or together with phosphorylation, occurs in real biological samples. This collection of data sets included patient, *in vivo*, and *in vitro* studies, and the research topics covered a wide range of biological phenomena (Table 3). In order to map tyrosine residues that are oxidized or both oxidized and phosphorylated, we used the MaxQuant/Andromeda software^30−32^ to identify proteins, peptides, and modification sites.

**Table 3:** Tyrosine Phosphorylation, Oxidation, and Tyrosine Oxidation with Phosphorylation in Phosphoproteomics Data by Variable Modification Search

						number of modified residues		
reference	ProteomeXchange data set (PXD) or MassIVE (MSV) ID^a^	sample type	research topic	raw files	identified peptide sequences	Ox-Y	p-Y	pOx-Y
Hoffman et al.^25^	PXD001543	patient samples: skeletal muscle	exercise	28	24 975	141	151	7
Kohtala et al.^26^		*in vivo*: mouse hippocampus	isoflurane anesthesia	6	7400	17	10	7
Lin et al.^27^	PXD017045	*in vivo*: mouse kidney	sepsis-induced kidney injury	18	10 240	19	2	4
Rahikainen et al.^28^		*in vitro*: mouse embryonic fibroblast cell line	cell adhesion signaling	12	9780	45	9	7
Sharma et al.^1^	PXD000612	*in vitro*: HeLa S3 cervical cancer cell line	phosphoproteomics method development and comparison of S, T, and Y phosphorylation	141	60 471	471	802	51
Tighe et al.^29^	MSV000083012^a^	*in vivo*: mouse lung	radiation-induced pulmonary fibrosis	18	4204	15	6	4

a The ProteomeXchange identifier of one data set could not be found.

In each data set, both oxidized tyrosine modifications and phosphorylated tyrosine modifications were identified with variable modification search in similar abundance and in numbers about 2 orders of magnitude lower than unmodified tyrosine residues in detected peptides. We also identified tyrosine residues that carried both oxidation and phosphorylation as a modification, albeit in low numbers (Table 3 and File S2). The same site phosphorylation and oxidation were rare and in the same order of magnitude as the stochastic probability of both oxidation and phosphorylation together. Due to the low numbers of identifications for tyrosine residues that are both phosphorylated and oxidized, false positives from the proteomics search engine cannot be ruled out. Therefore, we chose to further investigate the issue using a dependent peptide search and specific immonium ions.

Dependent peptide (DP) search is a ModifiComb^36^ implementation in MaxQuant, allowing the unrestricted search of peptide modifications, e.g., protein isoforms, cross-linking, and known and novel PTMs. It is false discovery rate controlled using a target decoy procedure separate from that of the main search and is therefore a suitable tool to search for novel and rare PTMs. DPs were searched alongside variable modifications, and DP modifications with a mass difference equal to phosphorylation and oxidation (+95.96 Da) with the highest localization in tyrosine residues were only found in the largest data set in which phosphotyrosines were specifically immunoenriched.^1^

In these DP results, 1223 individual modifications of 535 unique mass changes were observed and localized to tyrosine (Figure S5). Most of them (831 individual and 237 unique modifications) were within −100 and 100 Da and equal with highly abundant modifications such as phosphorylation, dephosphorylation, oxidation, and reduction. Notably, a modification with mass change equal to phosphorylation and oxidation (+95.96 Da) was detected (Figure S5).

**Figure 5:**
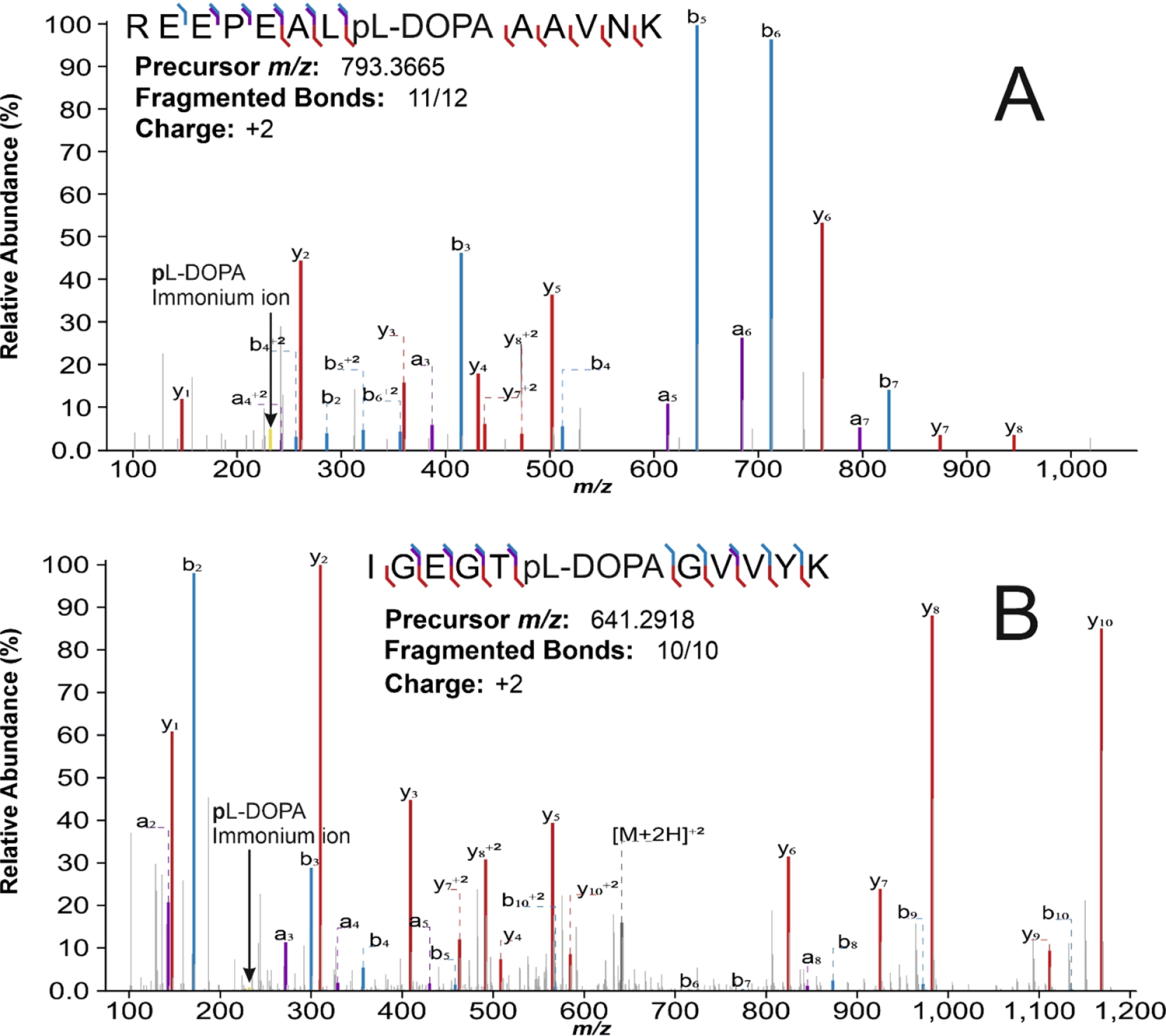
PSM examples with pl-DOPA and specific immonium ion. Two annotated MS/MS spectra from the DP search result (File S3). PSMs that have high confidence pl-DOPA (same site phosphorylation and oxidation of tyrosine) modification, high sequence fragment coverage, as well as specific pl-DOPA immonium ion ((A) 5.2% relative abundance, accurate mass of *m/z* 232.03825, <6 ppm mass accuracy and (B) 0.5%, *m/z* 232.03735, <2 ppm). Annotated MS/MS spectra are also available using USI: http://proteomecentral.proteomexchange.org/usi/?usi=mzspec:PXD000612:20120323_EXQ5_KiSh_SA_LabelFree_HeLa_pY_pervandate_rep1:scan:24850:REEPEALY[Oxidation][Phospho]AAVNK/2 and http://proteomecentral.proteomexchange.org/usi/?usi=mzspec:PXD000612:20120415_EXQ5_KiSh_SA_LabelFree_HeLa_pY_EGF5_rep1:scan:22556:IGEGTY[Oxidation][Phospho]GVVYK/2.

Tyrosine residues that carried both oxidation and phosphorylation as a modification were observed, again in low numbers. The same site phosphorylation and oxidation were matched to 22 MS/MS scans with PEP <0.01 in six peptides (File S3). This data set was rerun for DP without the same site phosphorylation and oxidation as a variable modification to remove these peptide spectral matches from the main or second peptide search and thus permitting unassigned MS/MS to be matched in DP search. This increased the matches to 99 MS/MS scans with PEP <0.01 in 19 peptides (File S3). Nine peptides were common between reanalyzed DP search and variable modification search, and reanalysis results included also PSMs of all peptides found in the first DP search (Figure S6). Some initial DP search PSMs with PEP >0.01 were decreased to PEP <0.01 after reanalysis. Of these peptides, five had the novel phospho-l-DOPA immonium ion (C_8_H_11_NO_5_P^+^, mass accuracy <10 ppm from exact mass *m/z* 232.03694, relative abundances from 0.5 to 7.8% with an average of 3.3%), 12 peptides had the known phosphotyrosine immonium ion (C_8_H_11_NO_4_P^+^, mass accuracy <10 ppm from exact mass *m/z* 216.04202, relative abundances from 0.5 to 30% with an average of 7.8%), and three peptides had both immonium ions in MS/MS. The annotated mass spectra of two PSMs with high sequence ion coverage and pl-DOPA immonium ion, unambiguously showing the pl-DOPA modification, are presented in Figure 5. All annotated MS/MS spectra are shown as universal spectral identifier (USI)^37^ along with the DP result information in File S3.

According to these results, tyrosine oxidation can be detected in various biological samples, in some rare cases together with phosphorylation on the same residue. Although false positives likely still persist, also excellent matches with sequence diagnostic ion series and pl-DOPA immonium ion peak were detected (Figure 5 and File S3). Given the previous research on endogenous tyrosine oxidation^38−42^ and the inhibitory role of tyrosine phosphorylation on its oxidation,^19^ as well as the findings presented in this work, tyrosine oxidation and its interplay with phosphorylation may be of biological significance.

Despite the fact that many of the peptide identifications with the variable modification search of both phosphorylated and oxidized tyrosine residues may be false positives, we decided to tentatively investigate the potential biological role of these modifications. Enrichment analysis of Gene Ontology^43,44^ terms was performed using Enrichr^45,46^ on the MaxQuant variable modification search results (proteins in which modified peptides were identified). Lists of proteins in which oxidized and oxidized and phosphorylated tyrosine residues were identified (File S2) were used as the input, and GO terms with adjusted *p*-value <0.01 for enrichment were considered significant. For both phosphorylation combined with oxidation and oxidation alone, no significantly enriched GO terms were shared between the data sets (File S2); rather, in studies where any GO terms were significantly enriched, they represented the sample type in the particular study. These results suggest that the potential biological significance of tyrosine oxidation and tyrosine oxidation and phosphorylation is not similar in different organisms, tissues, and biological functions but depends on the biological context.

## Discussion

Oxidative damage to proteins can cause protein cross-linking, fragmentation, and modification of amino acid residues, potentially resulting in inactive proteins. Oxidation of tyrosine residues mostly to protein-bound l-DOPA has the additional capacity to reduce, bind, and chelate transition metals and inflict secondary damage to other biomolecules by itself.^47^ While phosphorylation is one of the most prominent and investigated PTMs, little is known about the susceptibility of modified protein amino acid residues, such as oxidized tyrosine, to phosphorylation.

Here, we have shown that oxidized tyrosine residues (i.e., peptide-bound l-DOPA) can be phosphorylated, contrary to a previously postulated hypothesis,^17^ but the l-DOPA phosphorylation rate appears to be slower than with tyrosine. We also showed, using raw phosphoproteomics data from various experimental settings, that both tyrosine oxidation to l-DOPA and l-DOPA phosphorylation can be identified in biological samples, although in low numbers. As phosphorylated l-DOPA abundance is low, searching multiple variable modifications with low abundance can produce false positive and negative results.^36^ Thereby, complementary confirmation methods such as diagnostic ions or unrestricted search methods are required. The currently detected numbers of tyrosine residues carrying both phosphorylation and oxidation PTMs are orders of magnitude below phosphorylation or oxidation, and further research would benefit from efficient enrichment, MS/MS optimization for immonium ion formation, or ultradeep proteome approaches.

Based on bioinformatics enrichment analysis, these modifications may have biological functions, but the details of such functions remain to be elucidated. Recently, a proteome-wide spectral library of peptides with tyrosine residues enzymatically oxidized into l-DOPA was created for the data-independent acquisition (DIA) analysis of protein-bound l-DOPA.^48^ Given the low abundance of proteins with oxidized tyrosines, such tools are crucial for elucidating the biological role of tyrosine oxidation in proteins.

Phosphorylation of protein-bound l-DOPA instead of tyrosine could affect the stability of this prominent reversible PTM, protein folding, and interactions. Phosphorylated protein-bound l-DOPA could alter the tyrosine kinase signaling networks. The observed difference in phosphorylation rate could impede the dynamic nature of PTM-mediated and phosphorylation kinetics-sensitive signaling networks or oscillating phosphorylation systems such as those in circadian control.^49−51^

## Supporting Information Available

File S1: Summary of annotated MS/MS fragment information of peptides shown in Table 2 (XLSX)
File S2: Proteomics result tables (XLSX)
File S3: Dependent peptide search results (XLSX)
Extracted ion chromatograms (EICs) of nonphosphorylated and phosphorylated IR peptides in positive ion mode (Figure S1) and in negative ion mode (Figure S2); charge state distribution of IR peptides and phosphorylation states in positive ion mode (Figure S3); MS/MS total ion chromatograms of IR peptide phosphorylation products measured with DDA LC-MS/MS in positive ion mode (Figure S4); modification masses of tyrosine residues by dependent peptide search (Figure S5); Euler diagram of tyrosine phospho-oxidation peptides in proteomic searches (Figure S6); sample information (Table S1); exact masses, accurate masses, mass accuracies, and abundances of individual charge states of all charge states of phosphorylated IR peptides in positive ion mode (Table S2); deconvoluted IR peptide mass spectra in positive ion mode (Table S3); exact masses, accurate masses, mass accuracies, and deconvoluted accurate masses of phosphorylated IR peptides in negative ion mode (Table S4); and accurate masses, relative abundances (RA, in %), and mass accuracies (MA, in ppm) of phosphorylation diagnostic b and y ions shown in Table 2 (Table S5) (PDF)
