# Supplementary material for: Enzymatic Phosphorylation of Oxidized Tyrosine Residues
Source: J Proteome Res. 2023 May 5;22(6):1959–68. doi: 10.1021/acs.jproteome.3c00061 (PMC10243104; doi:10.1021/acs.jproteome.3c00061)
Supplement: Supplementary file 4 — pr3c00061_si_004.pdf [file pr3c00061_si_004.pdf]

Supporting information for

Enzymatic phosphorylation of oxidized tyrosine residues

Juho Heininen [1], Catharina Erbacher [2], Tapio Kotiaho [1, 3], Risto Kostiainen [1], Jaakko Teppo [1*]

[1] Drug Research Program and Division of Pharmaceutical Chemistry and Technology, Faculty of Pharmacy, P.O. Box 56, FI-00014 University of Helsinki, Helsinki, Finland

[2] Institute of Inorganic and Analytical Chemistry, University of Münster, Corrensstr. 48, 48149 Münster, Germany

[3] Department of Chemistry, Faculty of Science, P.O. Box 55, FIN-00014, University of Helsinki, Helsinki, Finland

Corresponding Author

*Email: jaakko.teppo@helsinki.fi. Tel. +358 503127719

Content

Supplementary figures....3

Figure S1. Extracted ion chromatograms (EIC) of non-phosphorylated and phosphorylated IR peptides in positive ion mode. 3

Figure S2. Extracted ion chromatograms (EIC) of non-phosphorylated and phosphorylated IR peptides in negative ion mode of the only found $[M-2H]^{2-}$ ion. 4

Figure S3. Charge state distribution of IR peptides and phosphorylation states in positive ion mode. 5

Figure S4. MS/MS total ion chromatograms of IR peptide phosphorylation products measured with DDA LC-MS/MS in positive ion mode. 6

Figure S5. Modification mass differences in tyrosine residues by dependent peptide search of Sharma dataset. 7

Figure S6. Euler diagram of phospho-oxidation tyrosine-residue peptides in proteomic searches in Sharma dataset. 8

Supplementary tables....9

Table S1. Sample information. Volumes of kinase buffer, kinase, individual IR peptides and ATP added. ..9

Table S2. Exact masses, accurate masses, mass accuracies and abundance of individual charge state of all charge states of phosphorylated IR peptides in positive ion mode. 10

Table S3. Deconvoluted IR peptide mass spectra in positive ion mode. 11

Table S4. Exact mass, accurate mass, mass accuracies and deconvoluted accurate mass of phosphorylated IR peptides in negative ion mode. 12

Table S5. Accurate masses, relative abundances (RA, in %) and mass accuracies (MA, in ppm) of phosphorylation diagnostic b and y ions shown in Table 2. 13

Supplementary spreadsheet files 14

File S1. Annotated MS/MS spectra. 14

File S2. Proteomics result tables. 14

File S3. Dependent peptide search results. 14

Supplementary figures

Figure S1. Extracted ion chromatograms (EIC) of non-phosphorylated and phosphorylated IR peptides in positive ion mode.

EIC as a sum of found charge states $[M+2H]^{2+}$ to $[M+4H]^{4+}$

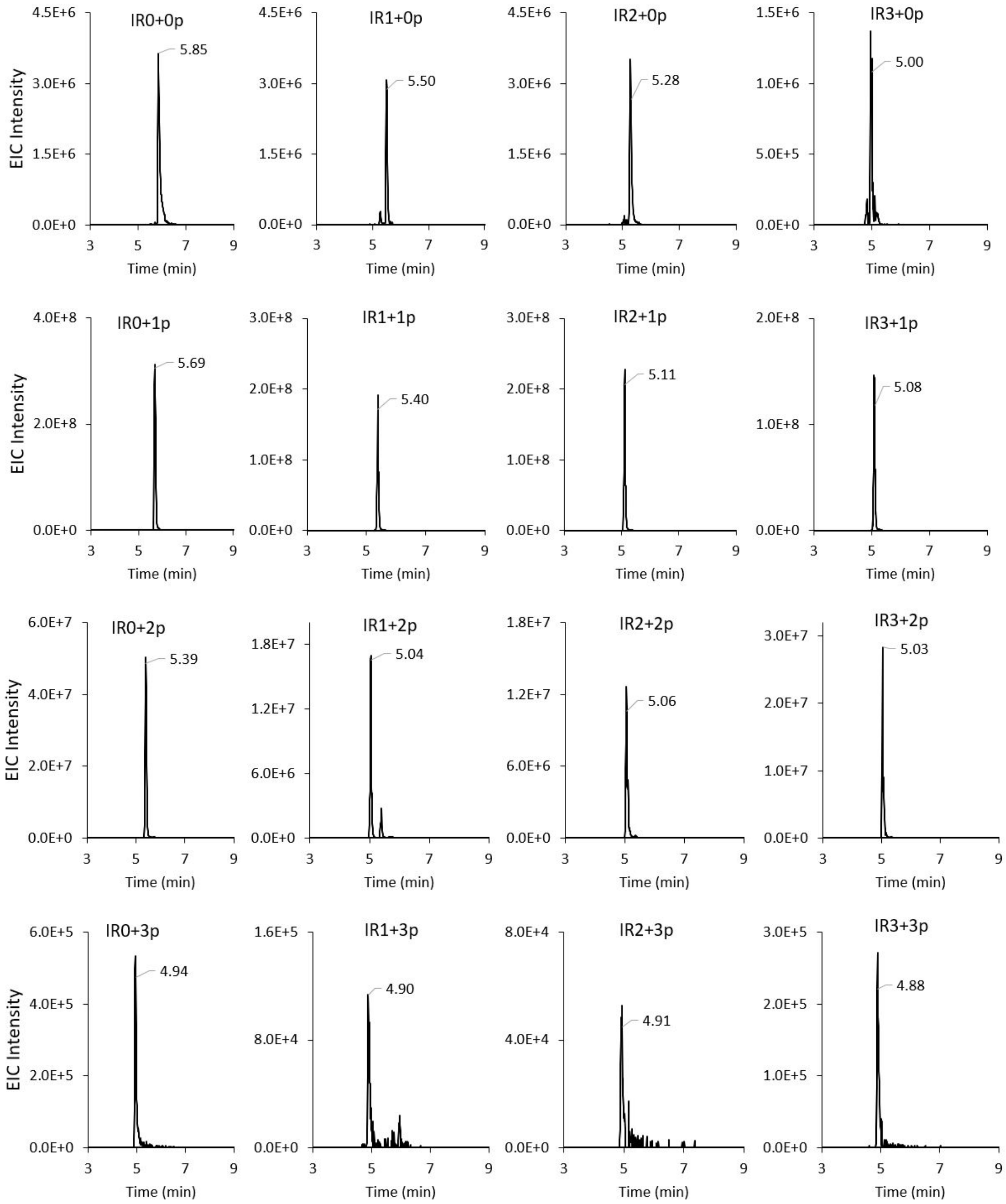

Figure S2. Extracted ion chromatograms (EIC) of non-phosphorylated and phosphorylated IR peptides in negative ion mode of the only found $[M-2H]^{2-}$ ion.

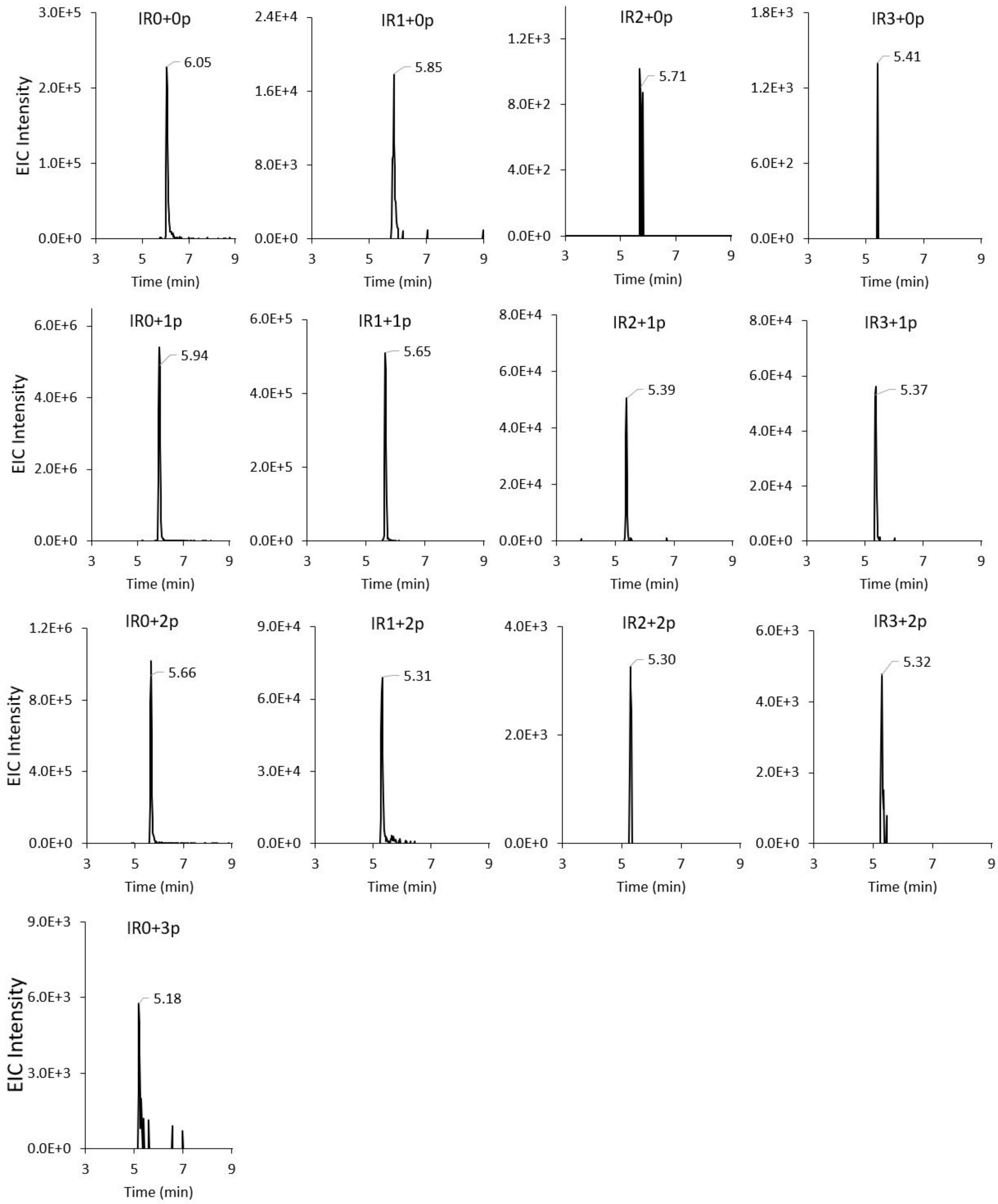

Figure S3. Charge state distribution of IR peptides and phosphorylation states in positive ion mode.

□=$[M+2H]^{2+}$, ■=$[M+3H]^{3+}$, ▩=$[M+4H]^{4+}$

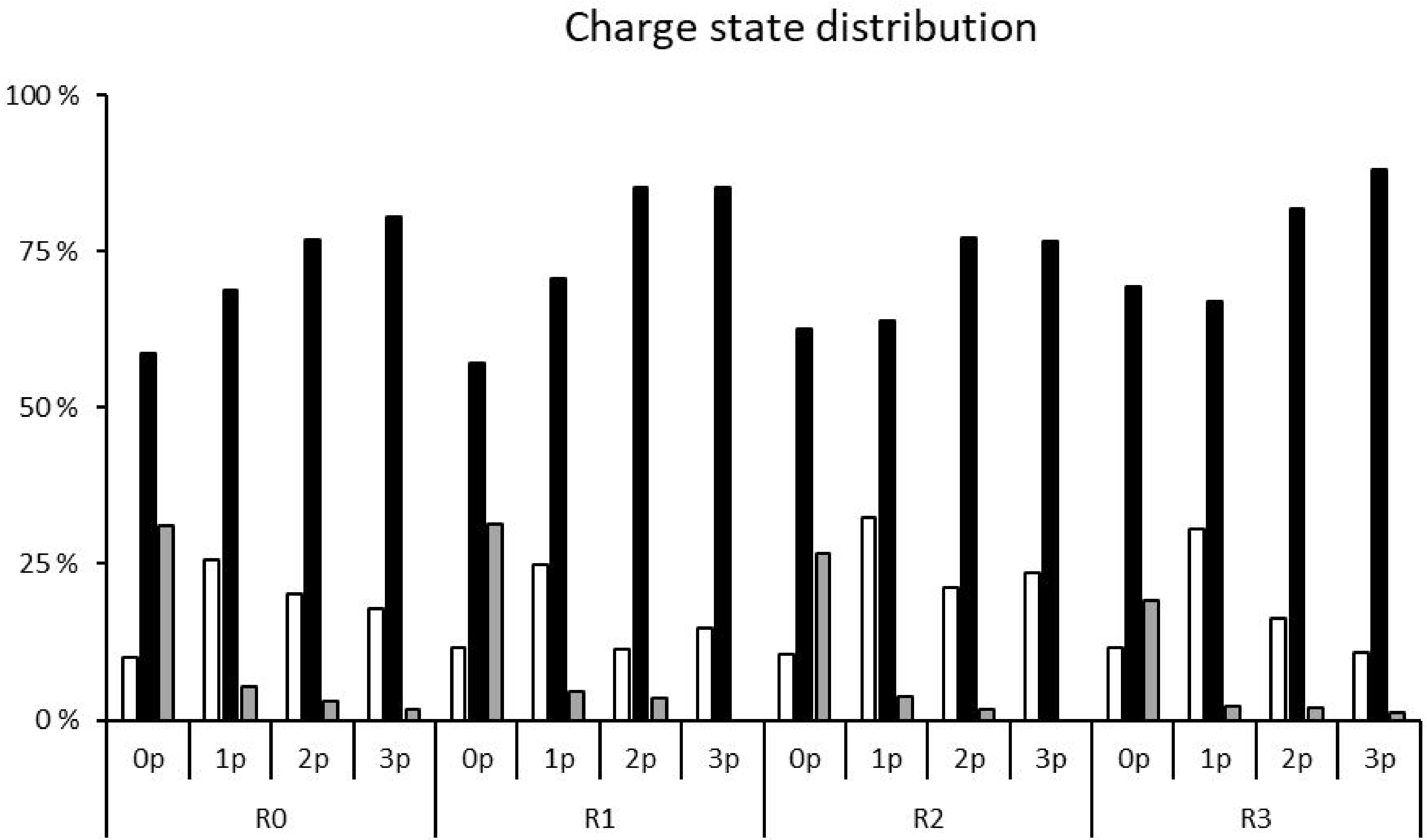

Figure S4. MS/MS total ion chromatograms of IR peptide phosphorylation products measured with DDA LC-MS/MS in positive ion mode.

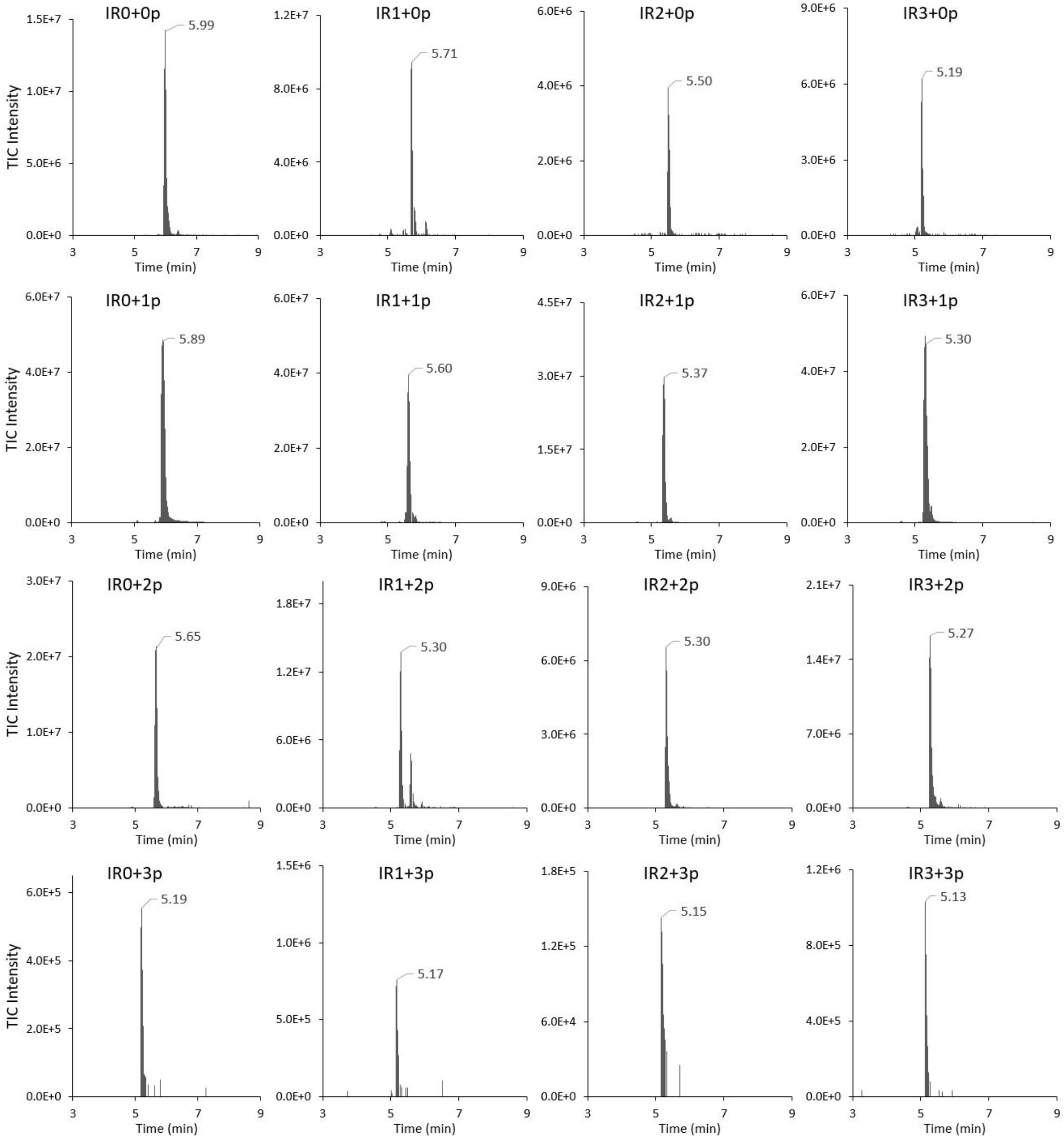

Figure S5. Modification mass differences in tyrosine residues by dependent peptide search of Sharma dataset.

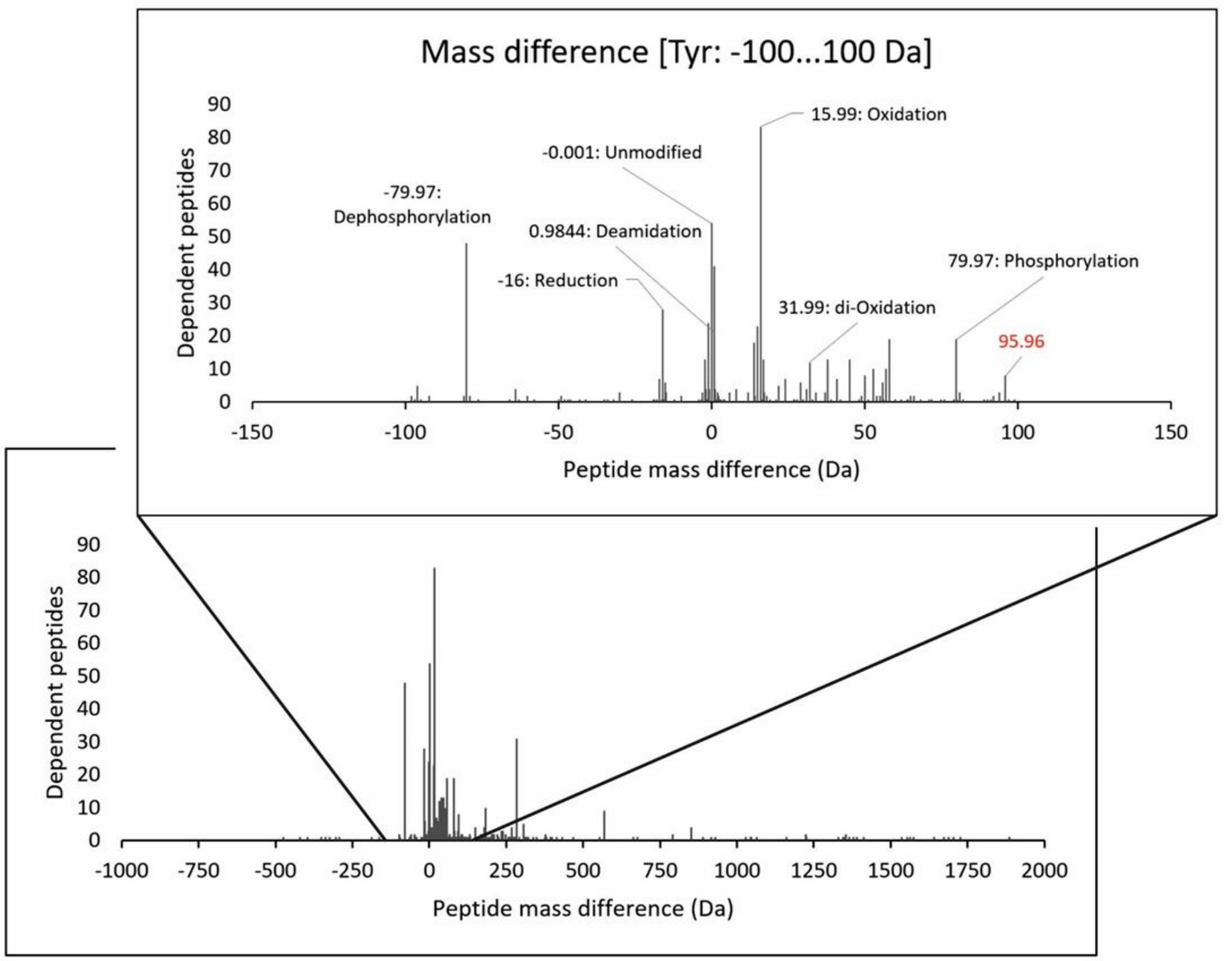

Figure S6. Euler diagram of phospho-oxidation tyrosine-residue peptides in proteomic searches in Sharma dataset.

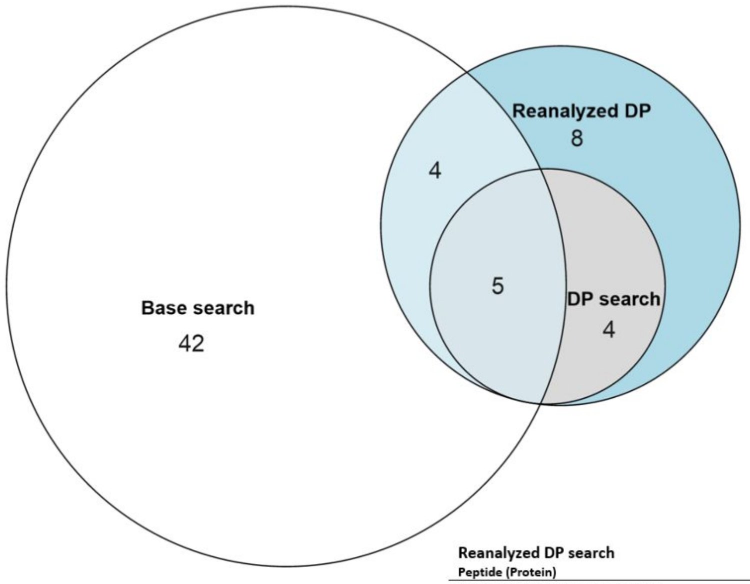

Base search

Peptide (Protein)

LGSSDILNY*SGNLMEPPFQNQK (O14530|TXND9)
ELEEIY*MLPR (O14646|CHD1;O14647|CHD2)
VEGDNIY*VR (O43491|E41L2)
LNGPQDHSHLLY*STIPR (O60716|CTND1)
SESVVY*ADIRK (O95297|MPZL1)
GSHQISLDNPDY*QQDFFPK (P00533|EGFR)
GSTAENAEY*LR (P00533|EGFR)
NINSINFDNPVY*QK (P01130|LDLR)
GGPGSAVSPY*PTFNPSSDVAALHK (P04083|ANXA1)
LIEDNEY*TAR (P06239|LCK)
LIEDNEY*TARQGAK (P06241|FYN;P12931|SRC;P07947|YES)
IGEGTY*GVVYK (P06493|CDK1;Q00526|CDK3;P24941|CDK2)
DELHIVEAEAMNY*EGSPIK (P06748|NPM)
LSLEGDHSTPPSAY*GSVK (P07355|ANXA2;A6NMY6|AXA2L)
LNY*QTPGMRDHPPIPITDLADNIER (P10586|PTPRF)
LDGENIY*IR (P11171|EPB41)
RVSVCAETY*NPDEEEEDTDPRVIHPK (P13861|KAP2)
DGDSYDPY*DFSDTEEEMPQVHTPK (P25205|MCM3)
VLEDDPEATY*TTSGGK (P29317|EPHA2)
QMPPPPPCPGRELFDDPSY*VNVQNLDK (P29353|SHC1)
IDEPSTPY*HSMMGDDEDACSDTEATEAMAPDILARK (P41236|IPP2)
LLLEGQGESGGGSLGAQPLLQPSHY*GQSGISMSHMDLR (P42226|STAT6)
QCLEDSDAGASNEY*DSSPAAWNK (P46063|RECQ1)
Y*PSPPMGSVSAPNLPTAEDNLEYVR (P46109|CRKL)
LGLAPQIQDLY*GK (P46940|IQGA1)
TYSECEDGTY*SPEISWHHR (P48651|PTSS1)
VGEEEHVY*SFPNK (P49023|PAXI)
MNEEY*DVIVLGTGLTECILSGIMSVNGKK (P50395|GDIB)
LGHVVMGNNAVSPY*QQVIEK (P60228|EIF3E)
NHTVQTFFSPVNSGQPPNY*EMLK (Q01628|IFM3;C9JQL5|DSA2D)
LALEAAQLDGPHFSCLY*PDGVFY*DLDSCK (Q01892|SPIB)
TPGRPLSSY*GMDSRPPMAIFELLDYIVNEPPPK (Q02750|MP2K1)
TPGRPLSSYGMDSRPPMAIFELLDY*IVNEPPPK (Q02750|MP2K1)
LCDFGSASHVADNDITPY*LVSR (Q13523|PRP4B)
VIQPHGGGSNPLEGTQHLQDVPY*VMVR (Q14126|DSG2)
DLHMERVISY*EEGK (Q15382|RHEB)
RPNY*KNVGEEEDEEK (Q2KHT3|CL16A)
NTFTAWSDEESDY*EIDDRDVNK (Q6PKG0|LARP1)
LSLHLY*GLGGLRPAPGATPR (Q6ZW31|SYDE1)
FQSSAVMALQEASEAY*LVGLFEDTNLCAIHAK (Q71DI3|H32)
SLLDGSGLVGY*AQEVLK (Q7Z4K8|TRI46)
TLEPVKPPTVPNDY*MTSPAR (Q8IZP0|ABI1)
MQTENLGVVYYVNKDFKNEY*K (Q8TBM8|DJB14)
IQAAEY*GLVVLEEK (Q96G01|BICD1)
YTEFY*HVPTHSDASK (Q96HC4|PDLI5)
LGEY*EDVSRVEK (Q99426|TBCB)
GRLDSSEMDHSENEDY*TMSSPLPGK (Q9NTI5|PDS5B)
REEPEALY*AAVNK (Q9NZM3|ITSN2)
HPDIY*AVPIK (Q9UDY2|ZO2)
TQSY*PTDWSDDESNNPFSSTDANGDSNPFDDDATSGTEVR (Q9UNF0|PACN2)
GY*DSEDDTWEPEQHLVNCEEYIHDFNR (Q9Y232|CDYL)

Reanalyzed DP search

Peptide (Protein)

REEPEALY*AAVNK (Q9NZM3|ITSN2)
HPDIY*AVPIK (Q9UDY2|ZO2)
IGEGTY*GVVYK (P06493|CDK1;P24941|CDK2;Q00526|CDK3)
SLY*PSSEIQLNNK (O75886|STAM2)
NVY*ATTVGTPYK (Q13439|GOGA4)
EENPY*ARFENN (P53801|PTTG)
LGLAPQIQDLY*GK (P46940|IQGA1)
VGEEEHVY*SFPNK (P49023|PAXI)
GSHQISLDNPDY*QQDFFPK (P00533|EGFR)
VIY*DFIEK (O00401|WASL)
IY*ESIEEAK (O75116|ROCK2)
LCDFGSASHVADNDITPY*LVSR (Q13523|PRP4B)
LSLEGDHSTPPSAY*GSVK (A6NMY6|AXA2L;P07355|ANXA2)
SESVVY*ADIRK (O95297|MPZL1)
LTIY*SEADLRK (Q15154|PCM1)
NINSINFDNPVY*QK (P01130|LDLR)
LQLDNQY*AVLENQK (Q9UKY7|CDV3)
LIEDNEY*TAR (P06239|LCK;P06241|FYN;P07947|YES;P12931|SRC)
ELFDDPSY*VNVQNLDK (P29353|SHC1)
GEPNVSY*ICSR (P49840|GSK3A;P49841|GSK3B)
LGEY*EDVSRVEK (Q99426|TBCB)

DP search

Peptide (Protein)

GSHQISLDNPDY*QQDFFPK (P00533|EGFR)
LCDFGSASHVADNDITPY*LVSR (Q13523|PRP4B)
IGEGTY*GVVYK (P06493|CDK1;P24941|CDK2;Q00526|CDK3)
VIY*DFIEK (O00401|WASL)
EENPY*ARFENN (P53801|PTTG)
REEPEALY*AAVNK (Q9NZM3|ITSN2)
SESVVY*ADIRK (O95297|MPZL1)
IY*ESIEEAK (O75116|ROCK2)
LTIY*SEADLRK (Q15154|PCM1)

Area proportional Euler diagram of peptides with phospho-oxidation tyrosine in Sharma dataset. Unique peptides with phospho-oxidation modified tyrosine and corresponding proteins found by variable modification search (Base search), dependent peptide (DP) search done alongside variable modification search and reanalyzed DP search. Euler diagram was done in R v4.2.1.using the eulerr library.

Supplementary tables

Table S1. Sample information. Volumes of kinase buffer, kinase, individual IR peptides and ATP added.

Sample	1x Kinase buffer A (µL)	INSR kinase 0.1 mg/mL (µL)	Peptide 1 mg/ml stocks	ATP 5 mg/ml, (µL)
1. A-eluent blank	150	10	20 µL of A-eluent	14
2. Peptide mix	150		5 µL of each	14
3. Control IR0	150		20 µL of IR0	14
4. Control IR1	150		20 µL of IR1	14
5. Control IR2	150		20 µL of IR2	14
6. Control IR3	150		20 µL of IR3	14
**Reaction rates:**				
7. 1 min with mix	150	10	5 µL of each	14
8. 5 min with mix	150	10	5 µL of each	14
9. 30 min with mix	150	10	5 µL of each	14
10. 60 min with mix	150	10	5 µL of each	14
**Individual samples:**				
11. IR0	150	10	20 µL of IR0	14
12. IR1	150	10	20 µL of IR1	14
13. IR2	150	10	20 µL of IR2	14
14. IR3	150	10	20 µL of IR3	14
15. Mix	150	10	5 µL of each	14

Table S2. Exact masses, accurate masses, mass accuracies and abundance of individual charge state of all charge states of phosphorylated IR peptides in positive ion mode.

IR0: TRDIYETDYYRK

IR1: TRDI(L-DOPA)ETDYYRK

IR2: TRDI(L-DOPA)ETDY(L-DOPA)RK

IR3: TRDI(L-DOPA)ETD(L-DOPA)(L-DOPA)RK

**Charge state**	**+2**				**+3**				**+4**			
**Peptide**	**Exact mass**	**Accurate mass**	**Abundance**	**Mass accuracy (mmu)**	**Exact mass**	**Accurate mass**	**Abundance**	**Mass accuracy (mmu)**	**Exact mass**	**Accurate mass**	**Abundance**	**Mass accuracy (mmu)**
IR0+0p	811.89408	811.89453	3.7E+5	0.45	541.59848	541.59875	2.1E+6	0.27	406.45068	406.45078	1.1E+6	0.10
IR0+1p	851.87723	851.87769	6.7E+7	0.46	568.25391	568.25537	1.8E+8	1.46	426.44226	426.44235	1.4E+7	0.09
IR0+2p	891.86038	891.86078	9.8E+6	0.40	594.90935	594.91052	3.7E+7	1.17	446.43383	446.43390	1.4E+6	0.07
IR0+3p	931.84353	931.84369	8.9E+4	0.16	621.56478	621.56500	4.0E+5	0.22	466.42541	466.42532	8.9E+3	-0.08
IR1+0p	819.89158	819.89221	3.5E+5	0.63	546.93015	546.93042	1.8E+6	0.27	410.44943	410.44949	9.6E+5	0.06
IR1+1p	859.87473	859.87500	4.3E+7	0.27	573.58558	573.58673	1.2E+8	1.15	430.44101	430.44101	7.6E+6	<0.01
IR1+2p	899.85788	899.85852	1.9E+6	0.64	600.24101	600.24298	1.4E+7	1.97	450.43258	450.43262	6.0E+5	0.04
IR1+3p	939.84103	939.84186	1.4E+4	0.83	626.89645	626.89648	8.1E+4	0.03	470.42416	not observed		
IR2+0p	827.88903	827.88965	3.7E+5	0.62	552.26178	552.26208	2.2E+6	0.30	414.44816	414.44815	9.4E+5	<0.01
IR2+1p	867.87218	867.87299	7.4E+7	0.81	578.91721	578.91864	1.5E+8	1.43	434.43973	434.43985	8.6E+6	0.12
IR2+2p	907.85533	907.85553	2.7E+6	0.20	605.57265	605.57275	9.8E+6	0.10	454.43131	454.43130	2.2E+5	<0.01
IR2+3p	947.83848	947.83862	1.2E+4	0.14	632.22808	632.22833	4.0E+4	0.25	474.42288	not observed		
IR3+0p	835.88648	835.88678	1.0E+5	0.30	557.59341	557.59338	6.2E+5	-0.03	418.44688	418.44690	1.7E+5	0.02
IR3+1p	875.86963	875.87030	3.7E+7	0.67	584.24885	584.25012	8.1E+7	1.27	438.43846	438.43840	2.8E+6	-0.05
IR3+2p	915.85278	915.85303	4.6E+6	0.25	610.90428	610.90417	2.3E+7	-0.11	458.43003	458.42999	5.3E+5	-0.04
IR3+3p	955.83593	955.83527	2.9E+4	-0.66	637.55971	637.56018	2.4E+5	0.47	478.42161	478.42209	3.0E+3	0.48

Table S3. Deconvoluted IR peptide mass spectra in positive ion mode.

Peptide	Exact mass	Deconvoluted accurate mass	Mass accuracy (mmu)
IR0+0p	1621.7736	1621.7743	0.70
IR0+1p	1701.7399	1701.7418	1.89
IR0+2p	1781.7062	1781.7077	1.53
IR0+3p	1861.6725	1861.6727	0.21
IR1+0p	1637.7686	1637.7694	0.77
IR1+1p	1717.7349	1717.7362	1.34
IR1+2p	1797.7012	1797.7036	2.45
IR1+3p	1877.6675	not observed	
IR2+0p	1653.7635	1653.7642	0.71
IR2+1p	1733.7298	1733.7319	2.13
IR2+2p	1813.6961	1813.6963	0.23
IR2+3p	1893.6624	not observed	
IR3+0p	1669.7584	1669.7586	0.19
IR3+1p	1749.7247	1749.7263	1.65
IR3+2p	1829.6910	1829.6910	0.00
IR3+3p	1909.6573	1909.6580	0.67

Table S4. Exact mass, accurate mass, mass accuracies and deconvoluted accurate mass of phosphorylated IR peptides in negative ion mode.

Peptide	Exact mass	Accurate mass	Abundance	Mass accuracy (mmu)	Deconvoluted accurate mass	Mass accuracy (mmu)
IR0+0p	809.87952	809.88190	2.3E+5	2.38	1621.77836	4.76
IR0+1p	849.86267	849.86487	5.4E+6	2.20	1701.74430	4.40
IR0+2p	889.84582	889.84821	9.4E+5	2.39	1781.71098	4.78
IR0+3p	929.82897	929.83154	5.0E+3	2.57	1861.67764	5.14
IR1+0p	817.87702	817.87927	1.8E+4	2.25	1637.77310	4.50
IR1+1p	857.86017	857.86230	4.7E+5	2.13	1717.73916	4.26
IR1+2p	897.84332	897.84583	6.9E+4	2.51	1797.70622	5.02
IR1+3p	937.82647	937.82898	7.7E+2	2.51	1877.67252	5.02
IR2+0p	825.87447	825.87677	9.1E+2	2.30	1653.76810	4.60
IR2+1p	865.85762	865.86005	5.1E+4	2.43	1733.73466	4.86
IR2+2p	905.84077	905.84283	3.3E+3	2.06	1813.70022	4.12
IR2+3p	945.82392	not observed				
IR3+0p	833.87192	833.87421	1.4E+3	2.29	1669.76298	4.58
IR3+1p	873.85507	873.85724	5.6E+4	2.17	1749.72904	4.34
IR3+2p	913.83822	913.84021	4.8E+3	1.99	1829.69498	3.98
IR3+3p	953.82137	not observed				

Table S5. Accurate masses, relative abundances (RA, in %) and mass accuracies (MA, in ppm) of phosphorylation diagnostic b and y ions shown in Table 2.

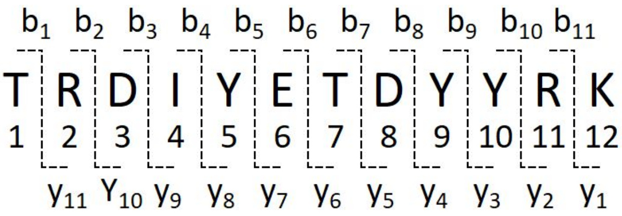

Peptide	b_4 Accurate mass	RA (%)	MA ppm	b_5 Accurate mass	RA (%)	MA ppm	y_3 Accurate mass	RA (%)	MA ppm	y_4 Accurate mass	RA (%)	MA ppm	y_5 Accurate mass	RA (%)	MA ppm
IR0+0p	486.2672	(62)	0.30	649.3307	(78)	0.54	466.2774	(59)	0.29	629.3409	(77)	0.49	744.3680	(69)	0.63
IR0+1p	486.2673	(65)	0.49	649.3312	(90)	1.30	466.2771	(35)	-0.37	629.3410	(1)	0.69	*726.3579*	(2)	1.32
										709.3078^{p}	(43)	1.27	824.3348^{p}	(52)	1.21
IR0+2p	486.2673	(57)	0.49	649.3308	(65)	0.64	466.2773	(11)	0.02	709.3074^{p}	(15)	0.75	824.3343^{p}	(17)	0.54
				729.2974^{p}	(13)	0.87	546.2440^{p}	(17)	0.71	789.2737^{pp}	(23)	0.57	904.3007^{pp}	(25)	0.54
IR0+3p	486.2671	(12)	0.05	649.3296	(1)	-1.24	546.2435^{p}	(7)	-0.08	**355.1569**^{p}	(2)	-0.64	*806.3215*^{p}	(2)	-2.16
				729.2962^{p}	(11)	-0.72				789.2731^{pp}	(10)	-0.13	904.3000^{pp}	(11)	-0.20
IR1+0p	486.2673	(59)	0.42	665.3257^{o}	(75)	0.67	466.2773	(57)	0.22	629.3409	(74)	0.59	744.3680	(71)	0.71
IR1+1p	486.2673	(69)	0.55	665.3261^{o}	(93)	1.22	466.2771	(37)	-0.30	629.3412	(2)	0.97	744.3683	(1)	1.12
										709.3078^{p}	(47)	1.27	824.3349^{p}	(59)	1.28
IR1+2p	486.2672	(38)	0.36	665.3256^{o}	(59)	0.49	546.2439^{p}	(17)	0.59	709.3072^{p}	(2)	0.41	824.3345^{p}	(2)	0.77
										789.2737^{pp}	(24)	0.65	904.3004^{pp}	(26)	0.27
IR1+3p	486.2670	(8)	-0.20	665.3263^{o}	(1)	1.50	546.2437^{p}	(5)	0.26	789.2726^{pp}	(4)	-0.82	904.2991^{pp}	(5)	-1.22
				745.2917^{op}	(6)	0.08									
IR2+0p	486.2672	(54)	0.24	665.3255^{o}	(68)	0.31	482.2722^{o}	(58)	0.16	645.3356^{o}	(71)	0.25	760.3624^{o}	(69)	-0.06
IR2+1p	486.2672	(66)	0.17	665.3259^{o}	(91)	0.95	482.2724^{o}	(36)	0.47	645.3361^{o}	(2)	0.91	760.3630^{o}	(1)	0.74
										725.3026^{op}	(45)	1.03	840.3295^{op}	(56)	0.86
IR2+2p	486.2671	(25)	0.11	665.3254^{o}	(38)	0.12	562.2385^{op}	(7)	0.10	**363.1546**^{op}	(2)	0.26	920.2950^{opp}	(11)	-0.10
										805.2679^{opp}	(10)	-0.31			
IR2+3p	486.2677	(4)	1.40	745.2925^{op}	(5)	1.19	not observed			**403.1380**^{opp}	(17)	0.81	**460.6513**^{opp}	(9)	0.35
IR3+0p	486.2671	(48)	-0.01	665.3254^{o}	(62)	0.12	482.2722^{o}	(48)	0.09	661.3304^{oo}	(62)	0.02	*758.3468*^{oo}	*(6)*	0.07
IR3+1p	486.2669	(16)	-0.27	665.3260^{o}	(23)	1.13	482.2722^{o}	(10)	0.22	**331.1688**^{oo}	(1)	<0.01	*758.3477*^{oo}	(2)	1.20
										741.2977^{oop}	(8)	1.30	856.3245^{oop}	(10)	1.03
IR3+2p	486.2672	(58)	0.24	665.3256^{o}	(74)	0.49	482.2722^{o}	(6)	0.16	741.2971^{oop}	(4)	0.66	776.3576^{oo}	(2)	0.38
				745.2921^{op}	(4)	0.57	562.2388^{op}	(15)	0.64	821.2635^{oopp}	(10)	0.63	856.3240^{oop}	(7)	0.54
													936.2903^{oopp}	(12)	0.33
IR3+3p	486.2669	(8)	-0.39	745.2914^{op}	(4)	-0.33	562.2383^{op}	(4)	-0.23	**371.1514**^{oop}	(1)	-1.50	856.3237^{oop}	(2)	0.26
										821.2625^{oopp}	(1)	-0.63	936.2899^{oopp}	(1)	-0.06

Superscript o= oxidation, oo= two oxidations, p= phosphorylation, pp= two phosphorylations

bold: Fragments with double charge

italics: Fragments with H_2O neutral loss

Supplementary spreadsheet files

File S1. Annotated MS/MS spectra.

Summary of annotated MS/MS fragment information of peptides shown in Table 2. Diagnostic a, b, x, and y - ions with charge states from +1 to +3 and neutral losses of H_2O and NH_3 of above 1% from average mass spectra of main chromatographic peaks were annotated using IPSA. Peptide, peptide specific oxidation site, phosphorylation state and site, precursor charge state, Modifications (modification type, modification location and mass change), exact m/z of precursor at charge state +3, Number of matched fragments in MS/MS spectra, corresponding number of annotated peptide bonds and percentage of annotated fragment abundance of total ion chromatogram (TIC) are shown.

This is in separate Supporting Information file

File S2. Proteomics result tables.

The tables in File S2 are separately presented for peptides with simultaneous same tyrosine phosphorylation and oxidation. All tables have been created from the MaxQuant search output files. The complete MaxQuant search output files have been uploaded to the MassIVE repository (https://massive.ucsd.edu/) with the identifier MSV000090106.

This is in separate Supporting Information file

File S3. Dependent peptide search results.

The tables in File S3 are separately presented for DP search along standard variable modification base search and DP reanalysis. From this data, clusters with cluster mass of 95.96 Da and localization probability highest with tyrosine were parsed to individual peptide-spectrum matches (PSMs). Results with posterior error probability (PEP) >0.01 are greyed out. Universal spectrum identifier (USI) is written for all results and individual MS/MS are visible using from
http://proteomecentral.proteomexchange.org/usi/?usi=

The complete MaxQuant search output files have been uploaded to the MassIVE repository (https://massive.ucsd.edu/) with the identifier MSV000090106.

This is in separate Supporting Information file
